# Antioxidants Properties of Natural Products: A Themed Issue in Honor of Professor Isabel C.F.R. Ferreira

**DOI:** 10.3390/antiox9040286

**Published:** 2020-03-28

**Authors:** Lillian Barros

**Affiliations:** Centro de Investigação de Montanha (CIMO), Instituto Politécnico de Bragança, Campus de Santa Apolónia, 5300-253 Bragança, Portugal; lillian@ipb.pt

Professor Isabel C.F.R. Ferreira has worked on the Chemistry of Natural Products, specifically the extraction, purification, and stabilization of myco and phytochemicals; development of natural ingredients, nutraceuticals, and innovative functional food formulations. She has developed numerous methodologies for the extraction, refinement, and chemical characterization of bioactive molecules from mushrooms and plants, and also improved the analytical methodologies of different biomolecules. She has prospected more than 300 species and discovered natural sources of ingredients with different capacities: bioactive, preservative, and coloring. In 2007, she led the research leading to the discovery that Portuguese wild mushrooms are powerful sources of compounds with bioactive properties. Subsequently, she showed that not only wild, but also commercial mushrooms are sources of bioactive compounds and equilibrated nutrients, leading to the first studies on species from Serbia, Ghana, Brazil, China, and Argentina, among others. In 2012, Isabel C.F.R. Ferreira discovered that specific mushroom species have significant antitumor, antimicrobial, and anti-inflammatory activities that can be improved modifying chemical substituents in specific molecules identified in those species. 

In 2009, she began prospecting plant species and discovered specific parts of vegetables, flowers, and medicinal plants that are unique sources of bioactive molecules, particularly in the class of phenolic compounds. Subsequent to this, she showed for the first time that synergistic effects can be achieved by mixing defined species and that in vitro culture under optimized conditions can increase the concentration of these compounds. She has also developed novel cheminformatic tools and models to predict bioactive properties and organize molecule databases, and has carried out docking studies on bioactive cellular targets. She has been working with many mushroom and plant producing companies, and in 2011 she started to study physical conservation methods, discovering that gamma rays and electron-beams can decontaminate the mentioned raw materials without changing their nutritional/chemical profile, and also improve the extraction of bioactive molecules. She also discovered that modified atmospheres can recover the use of traditional plant species. 

More recently, Professor Isabel C.F.R. Ferreira developed, improved, and optimized several extraction methodologies for different natural molecules with bioactive, coloring, and preservative capacities to be used as natural food ingredients/additives, and also their stabilization. With these stabilized ingredients, she has developed innovative food and cosmetic products (registered patents) with plant and mushroom-origin preservatives, colorants, and bioactives. Isabel F.C.F. Ferreira revision articles on bioactive molecules/properties of mushrooms and plants, natural preservatives, colorants, and conservation technologies, which are some of the most highly cited papers in the field.

The research performed by Professor Isabel C.F.R. Ferreira has placed her in top positions, being listed as a Highly Cited Researcher since 2015. She has published more than 675 articles and several innovative patents, supervised many students, and has been the principal researcher of numerous national and international projects. She has also gained a range of awards for her impact in Food Chemistry, for her advancement of research in Portugal, and for her outstanding work as one of the country’s top women in science. Now, she is working for the Portuguese Government as Secretary of State for Interior Valorization.

The special issue in honor of Professor Isabel C.F.R. Ferreira, with the thematic “Antioxidants Properties of Natural Products” has twenty-three original research or review articles. Within the reviews performed, Rusu et al. [1] revised the clinical trials and cohort studies published in the last decade on the influence of antioxidant diets on nuts and peanuts in preventing or delaying age-related illnesses in middle-aged and elderly individuals. Meanwhile, Afonso et al. [2] reviewed the use of brown macroalgae, considered as safe for consumption in Europe, as a valuable ingredient namely for the development pf phaeophyta-enriched food products. Gomez-Zavaglia et al. [3] reviewed the up to date literature on the chemical composition and bioactive effects of seaweeds, and reported a critical discussion about their potential as natural sources of new functional ingredients. Root vegetables as an alternative source of natural red–purple color ingredients for pharmaceutical or food applications were extensively revised by Spyridon and contributors [4]. Furthermore, a discussion of the main bioactivities of these potential sources of natural colorants were also explored by the authors. Additionally, Di Gioia et al. [5] continued this study (part 2) and revised the health effects of red–purple vegetables, including leafy vegetables, fruits, and others, in which the chemical composition and bioactivities of the investigated matrices were discussed. Finally, the revision performed by Balakrishman et al. [6] provides insight into the major components of *Murraya koenigii* (L.) Sprengel, and their pharmacological activities against different pathological conditions. 

Regarding the original research works performed, Reyes-Fermín et al. [7] evaluated α-mangostin, a phytochemical found in *Garcinia mangostana* L., regarding its antioxidant action in the preservation of mitochondrial function against *cis*-dichlorodiammineplatinum II induced damage using in vitro assays. Moreover, the antioxidant activity during the seven stages of development of *Amaranthus caudatus* was determined by Kamarać et al. [8], through several assays (ABTS•^+^, DPPH•, O2•^−^ scavenging activity, FRAP, and Fe^2+^ chelating ability). In addition, the phenolic profile of the plant in each stage was also described by the authors. Petropoulos et al. [9] performed the nutritional and chemical composition evaluation of different parts of *Portulaca oleracea* L., namely stems and leaves, harvested in three growth stages. Additionally, the cytotoxicity of these samples was also assessed. In the research performed by Molina and contributors [10], the *Lonicera caerulea* L. berry was assessed in terms of nutritional and chemical composition and bioactive properties, namely antioxidant and antimicrobial activities. Also, the stabilization of the anthocyanins from the fruit juice was accomplished by microencapsulation. 

Tanase et al. [11] characterized *Fagus sylvatica* L. bark in terms of phytochemical profile and bioactive potential. In addition, the improvement of the phenolic compounds extraction by microwave assisted extraction was carried out using a design experiment approach. Hou et al. [12] optimized the extraction of flavonoids from *Pteris cretica* L. by ultrasound-assisted extraction through a Response Surface Methodology. Furthermore, the identification of the major compounds was performed by chromatographic analysis, and the optimal extract was evaluated in terms of its antioxidant potential. Añibarro-Ortega et al. [13] characterized the nutritional composition, organic acids, and phenolic compounds of *Aloe vera* leaf (fillet, mucilage, and rind) and flowers, and their bioactive features, in order to enhance the exploitation of this plant as a valuable source of compounds for food and pharmaceutical industries application. Biosynthesis of silver nanoparticles (AgNP) using *Fagus sylvatica* L. bark polyphenolic extract was accomplished by Tanase et al. [14] and the obtained AgNPs were explored in terms of their antioxidant and antimicrobial activities. Rusu et al. [15] obtained bioactive rich molecules from the by-products of *Corylus avellana* L. (hazelnut involucre) using optimized conditions. The extract was evaluated through in vitro antioxidant tests and enzymatic inhibitory assays. Additionally, the cytotoxic and antioxidant effects of this extract was evaluated on two tumor cell lines and one normal cell line. Medrano-Padial et al. [16] evaluated the cytotoxic activity of a stilbenes-rich extract obtained from grapevine shoots on two tumor cell lines, with the aim of providing more evidence on the use of this kind of phytochemicals in wine preservation. Pereira-Maróstica and coauthors [17] studied the capability of methyl jasmonate to reduce articular and hepatic inflammation and oxidative stress in rats with adjuvant-induced arthritis. In the report performed by Lall et al. [18], the nutritional profile of *Sideritis perfoliata* L. was described. In addition, interesting bioactivities, such as anti-tyrosinase, antibacterial, and anti-elastase potential were evaluated to support the use of this plant as a potential cosmetic ingredient. The influence of cultivars and maturation stage on the phenolic composition of *Lycium barbarum* L. fruits were determined by Mocan and contributors [19]. Additionally, the authors correlated the phytochemical profiles obtained from the fruit extracts with the antioxidant and anti-fungal activities, as also, with tyrosinase inhibition. Molecular mechanisms involved in the biological effects of quercetin were studied by Ayuda-Durán et al. [20], using *Caenorhabditis elegans* as a model organism with different methodological approaches. Lee et al. [21] assessed the 3,3′-Diindolylmethane, a metabolite of indole-3-carbinol present in Brassicaceae vegetables, regarding its potential for prevention and therapy of neurodegenerative diseases. The authors evaluated in vitro effects of this compound against oxidative stress-induced issues. In order to provide a means for the disposal and valorization of walnut leaves, a phenolic-rich extract with high antioxidant activity from this matrix was obtained by Fernández-Agulló et al. [22], under optimized conditions for a maceration extraction system. Finally, Cásedas et al. [23] surveyed the neuroprotective action of urolithin A, as a gastrointestinal metabolite from ellagitannins, against oxidative-stress provoked in neuro-2a cells.

Overall, this special issue includes research related to the work that Professor Isabel C.F.R. Ferreira has been performing across her career, which is to exploit natural products, including natural compounds obtained from plants, mushrooms, marine, bee products, among others, focusing on the structural characterization and/or isolation of compounds with bioactivity; chemical, biological, or biochemical methods for the evaluation of *in vivo* and in vitro bioactivity; clinical and nutritional trials focused on the bioactive properties of compounds, and elucidation of the mechanisms of action of bioactive compounds. 

May this themed issue be able to honor someone who, one day, proudly stated: “Never giving up is my helm, and the pleasure of discovery is my anchor”. A motivation to keep in mind in science, as in life.
